# Head and neck dermatitis is exacerbated by *Malassezia furfur* colonization, skin barrier disruption, and immune dysregulation

**DOI:** 10.3389/fimmu.2023.1114321

**Published:** 2023-02-22

**Authors:** Howard Chu, Su Min Kim, KeLun Zhang, Zhexue Wu, Hemin Lee, Ji Hye Kim, Hye Li Kim, Yu Ri Kim, Seo Hyeong Kim, Wan Jin Kim, Yang Won Lee, Kwang Hoon Lee, Kwang-Hyeon Liu, Chang Ook Park

**Affiliations:** ^1^ Department of Dermatology, Severance Hospital, Cutaneous Biology Research Institute, Yonsei University College of Medicine, Seoul, Republic of Korea; ^2^ Brain Korea 21 PLUS Project for Medical Science, Yonsei University College of Medicine, Seoul, Republic of Korea; ^3^ Brain Korea 21 FOUR Community Based Intelligent Novel Drug Discovery Education Unit, College of Pharmacy and Research Institute of Pharmaceutical Sciences, Kyungpook National University, Daegu, Republic of Korea; ^4^ Department of Dermatology, Myongji Hospital, Goyang, Republic of Korea; ^5^ Department of Dermatology, Konkuk University School of Medicine, Seoul, Republic of Korea

**Keywords:** atopic dermatitis, head and neck dermatitis, *Malassezia*, LC-MS/MS, lipid analysis, ceramide, red face syndrome

## Abstract

**Introduction & objectives:**

Head and neck dermatitis (HND) is a refractory phenotype of atopic dermatitis (AD) and can be a therapeutic challenge due to lack of responsiveness to conventional treatments. Previous studies have suggested that the microbiome and fungiome may play a role in inducing HND, but the underlying pathogenic mechanisms remain unknown. This study aimed to determine the link between HND and fungiome and to examine the contribution of *Malassezia furfur*.

**Materials and methods:**

To identify the effect of the sensitization status of *M. furfur* on HND, 312 patients diagnosed with AD were enrolled. To elucidate the mechanism underlying the effects of *M. furfur*, human keratinocytes and dermal endothelial cells were cultured with *M. furfur* and treated with Th2 cytokines. The downstream effects of various cytokines, including inflammation and angiogenesis, were investigated by real-time quantitative PCR. To identify the association between changes in lipid composition and *M. furfur* sensitization status, D-squame tape stripping was performed. Lipid composition was evaluated by focusing on ceramide species using liquid chromatography coupled with tandem mass spectrometry.

**Results:**

Increased sensitization to *M. furfur* was observed in patients with HND. Additionally, sensitization to *M. furfur* was associated with increased disease severity in these patients. IL-4 treated human keratinocytes cultured with *M. furfur* produced significantly more VEGF, VEGFR, IL-31, and IL-33. IL-4/*M. furfur* co-cultured dermal endothelial cells exhibited significantly elevated VEGFR, TGF-β, TNF-α, and IL-1β levels. Stratum corneum lipid analysis revealed decreased levels of esterified omega-hydroxyacyl-sphingosine, indicating skin barrier dysfunction in HND. Finally, *M. furfur* growth was inhibited by the addition of these ceramides to culture media, while the growth of other microbiota, including *Cutibacterium acnes*, were not inhibited.

**Conclusions:**

Under decreased levels of ceramide in AD patients with HND, *M. furfur* would proliferate, which may enhance pro-inflammatory cytokine levels, angiogenesis, and tissue remodeling. Thus, it plays a central role in the pathogenesis of HND in AD.

## 1 Introduction

Atopic dermatitis (AD) is a chronic relapsing pruritic eczematous skin disorder (1). It is considered a multifactorial disease in which allergen-induced immunoglobulin (Ig) E is thought to be one of the contributing factors, as the levels of these specific IgE antibodies are found to be increased in the sera of patients with AD (2). Head and neck dermatitis (HND) or red face syndrome is one of the features of AD, with characteristic diffuse erythema of the face, which is more commonly observed in infants and adults (3). Due to its chronicity and frequent relapses, patients’ quality of life is severely affected.

AD patients with HND have been found to be more sensitized to *Malassezia furfur* (*M. furfur*) and have increased levels of specific IgE compared to AD patients without HND (4–6). In addition, studies have reported that antifungal agents improve symptoms in these patients (3, 7), further supporting the association of *M. furfur* with HND in AD. However, the mechanisms underlying the development of HND and its association with *M. furfur* remain unclear.

*M. furfur* is a lipophilic yeast that constitutes the normal flora of the skin and produces lipases that break down sebum lipids into unsaturated fatty acids, oleic acid, and arachidonic acid (8). These fatty acids have desquamative effects on keratinocytes and induce the production of pro-inflammatory cytokines (9). *M. furfur* is associated with various cutaneous conditions, such as seborrheic dermatitis and tinea versicolor. Although these conditions may recur and become chronic, they usually respond well to treatment, unlike HND in AD patients. Therefore, we aimed to explore the role of *M. furfur* in the development of HND in AD.

## 2 Materials and methods

### 2.1 Ethical approval

This study was approved by the Institutional Review Board of Yonsei University Severance Hospital (IRB no. 4-2018-0334).

### 2.2 Selection of Patients with HND for basal characteristics analysis

For clinical evaluation of HND patients, a database of approximately 5,007 patients with AD who visited Yonsei University Severance Hospital’s Department of Dermatology in 2011 was reviewed retrospectively (10). A query search of patients’ electronic medical records with keywords “red face syndrome,” “red,” “redface,” and “Head and Neck Dermatitis” in both English and Korean was also performed.

### 2.3 Patient selection for the evaluation of *M. furfur* sensitization status

A total of 312 patients were diagnosed with AD at the Department of Dermatology, Severance Hospital, Yonsei University College of Medicine. The diagnosis was made according to the Hanifin and Rajka diagnostic criteria (11). Patients’ age and sex were also noted. The severity of AD was assessed using the eczema area and severity index (EASI). Patients with diffuse erythematous patches on the facial skin were categorized into the HND group, whereas the remaining subjects were categorized to the non-HND group. The patients were categorized according to different age groups in which childhood was defined as age less than 12 years, adolescents were aged between 12 and 18 years, and adults were categorized as those older than 18 years. Sensitization to *M. furfur* was assessed using CAP immunoassay. Sensitized status to *M. furfur* was defined by elevation in the levels of IgE specific to *M. furfur* above 0.70 kU/L (Class 2).

### 2.4 Histological evaluation of facial skin samples

For histological evaluation of HND, a histological database at the Yonsei University Severance Hospital was utilized. A query search of AD patients from 2011 who underwent facial skin biopsy was performed, and five patients were randomly selected from 9 candidates.

Histological analysis of non-HND face specimens was performed through a query search of AD patients who underwent skin biopsy on the face from 2013 for suspected concomitant vitiligo (usually the biopsy is conducted with non-lesional normal skin and lesional skin with vitiligo to compare the melanocyte population). Crude age filtering was performed to age-match AD patients. Among the 10 candidates, five patients were randomly selected for image analysis.

At 200x magnification, the longest distance from the subcorneal level to the basal layer was chosen arbitrarily for epidermal thickness after calibrating the scale bar to pixels. The number of vessels/mm^2^ was counted in the dermis of each slide section within a 100 µm distance from the epidermal–dermal junction.

Immunohistochemical staining was performed using paraffin-embedded sections with antibodies against factor VIII-related antigen (1:100, ab236284, Abcam), stromal cell-derived factor-1-alpha (SDF1-α) (1:100, ab25117, Abcam, Cambridge, United Kingdom), Interleukin-1-beta (IL-1-β) (1:100, ab2105, Abcam), tumor necrosis factor-alpha (TNF-α) (1:50, ab1793, Abcam), transforming growth factor-beta (TGF-β) (1:100, ab66043, Abcam), and vascular endothelial growth factor (VEGF) (1:200, ab1316, Abcam). Staining intensity was determined at 400x magnification at a randomly chosen area of the upper dermis. Images were quantified using ImageJ analysis tools (National Institutes of Health, Bethesda, MA).

To calculate the stained area of the antibody, we converted the original image to an 8-bit grayscale image (ImageJ>Image>8-bit), applied a binary threshold, and calculated the percentage positive for the stained part in the standard image. Quantification was performed relative to the entire selected region. The threshold for each staining was set as the average threshold of multiple immunostaining analyses performed by three independent experimenters.

### 2.5 Co-culture of organisms with human primary cells

Primary normal human epidermal keratinocytes were cultured at 37°C in 5% CO_2_ in Epilife medium supplemented with human keratinocyte growth supplement (Gibco, USA). Human microvascular endothelial cells (HMVECs) were cultured at 37°C in 5% CO_2_ in EBM-2 basal medium supplemented with EBM-2 growth medium (Lonza, USA).

*M. furfur* (ATCC 12078) was cultured at 30°C on Difco YM agar supplemented with 1% olive oil. *S. epidermidis* (*Staphylococcus epidermidis*, ATCC 12228) was cultured at 37°C on Difco tryptic soy agar. *C. acnes* (*Cutibacterium acnes*, ATCC 6919) was cultured at 37°C on forced clostridial medium (CM0149; Oxoid) with 2% agar. To induce hypoxia, a BD GasPakTM EZ Pouch was used. All the media were sterilized by autoclaving at 121°C for 15 min.

Organisms were harvested by centrifugation, and the pellet was suspended in the corresponding media. The organisms were heat-killed by incubation at 80°C for 3 min, and then co-cultured with normal human epidermal keratinocytes or human microvascular endothelial cells for 24 h at a density of 1 × 10^5^ cells/mL. To induce allergic environments, recombinant thymic stromal lymphopoietin (TSLP) (50 ng/mL) or IL-4 (50 ng/mL) was used.

### 2.6 Real-time quantitative PCR

The cells were harvested using trypsin-EDTA (0.25%) and centrifuged. Total RNA was extracted using the RNeasy Plus Mini Kit (Qiagen, Germany), following the manufacturer’s instructions. Next, cDNA was generated using a Veriti thermal cycler (Applied Biosystems). Real-time quantitative PCR was performed with cDNA supplemented with the appropriate TaqMan primers using a StepOnePlus PCR system (Applied Biosystems). mRNA expression level was calculated using the 2-ΔΔCT method. The primers used are as follows; VEGF (vascular endothelial growth factor) (Hs00900055_m1), VEGFR (VEGF receptor) (FLT1; Hs01052961_m1), IL-31 (Hs01098710_m1), IL-33 (Hs00369211_m1), TGF-β (Hs00998133_m1), TNF-α (Hs00174128_m1), IL-1β (Hs01555410_m1), and GAPDH (Hs02786624_g1).

### 2.7 Lipid extraction and quantification of human stratum corneum

The human stratum corneum was collected using D-squame tape (22 mm in diameter; CuDerm, Dallas, Tex) from the facial skin of five subjects from both HND and non-HND groups who provided informed consent. An additional number of five people who did not have any underlying disease history including dermatological conditions were recruited as the control group. Six consecutive D-squame tape strips were collected from each group. The first tape disc was discarded and the remaining tape discs were placed in separate tubes. The tapes were vortexed in 5 mL of methanol for 30 s. The tape debris was removed immediately, and the methanol solution was dried under a nitrogen stream at 30°C.

Total lipid extracts were separated into three fractions using silicic acid column chromatography: neutral lipids (TAG), free fatty acids, and ceramides. Half of the dried extracts were reconstituted in 200 μL of chloroform and loaded into silicic acid columns that were preconditioned with chloroform. After sample loading, each column was washed with 20 mL of chloroform to elute the neutral lipids, 20 mL of chloroform containing 0.2% acetic acid to elute free fatty acids, and 30 mL of methanol to elute the ceramides.

The fatty acid composition of SC lipid fractions was analyzed after derivatization (saponification and methylation) using a gas chromatograph equipped with an SPB-5 capillary column (5% phenol, 30 m, 0.25 mm ID, film thickness: 0.25 μm). The GC operating conditions were as follows: injector temperature, 305°C; detector temperature, 310°C FID; oven temperature, 175°C; 5°C/min; 300°C (20 min); and carrier gas, He.

The TAG and ceramide fractions of SC lipids were further analyzed using LC-MS/MS for profiling (12). Skin lipids were analyzed using a Nexera2 LC system connected to a triple quadruple mass spectrometer (LCMS 8060; Shimadzu, Kyoto, Japan) with a reversed-phase Kinetex C18 column (100 × 2.1 mm, Phenomenex, Torrance, CA, USA). Mobile phases were water/methanol mixture (1:9, *v*/*v*) with 10 mM ammonium acetate (A) and isopropanol/methanol mixture (5:5, *v*/*v*) with 10 mM ammonium acetate (B). The gradient elution was performed as follows: 0 min (30% of B), 0–15 min (95% of B), 15–20 min (95% of B), and 20–25 min (30% of B). Quantitation was conducted by selected reaction monitoring (SRM) of the [M + H]^+^ or [M + NH_4_^+^] ion and related product ion for each lipid species. The concentration of each target lipid species was calculated as the ratio of the target analyte to the internal standard (IS) multiplied by the concentration of the IS. Single-point calibrations of each target lipid species were conducted using a selected IS for each lipid class (NS(d18:1/12:0), NdS(d18:0/12:0), NP(t18:0/8:0), AS(d18:1/18:1), AdS(d18:0/12:0), AP(t18:0/6:0), E(18:2)O(16)S(18), E(18:2)O(16)P(18), A(18:1)NS(d18:1/17:0), A(18:1)NS(d18:1/17:0), sphingosine d_9_, OP(t18:0/16:0), OP(t18:0/16:0), OP(t18:0/16:0), and TG 45:0(15:0/15:0/15:0) for NS, NdS, NP, AS, AdS, AP, EOS, EOP, 1-*O*-Acyl-NS, 1-*O*-Acyl-AS, long chain base (LCB), OS, OP, OH, and TG, respectively).

### 2.8 Assessment of the effect of ceramide on *M. furfur* colonization

Lyophilized ceramide (esterified omega-hydroxyacyl-sphingosine; EOS) was purchased from Avanti Polar Lipids (USA). EOS was added to sterilized media at 45°C and placed on a magnetic stirrer. *M. furfur* and *C. acnes* were cultured on appropriate culture media discs, and their colonization patterns were observed. The concentrations of ceramide (EOS) were as follows: control (no additional treatment), 2, 5, and 10 μg/mL. Colony area analysis was performed using ImageJ software (13). The observed colony area values were divided by the colony area of the control (no EOS) and compared between different EOS treatments.

*Statistical Analysis*

The Student’s t-test was used for dependent samples, and the nonparametric Mann–Whitney U test was used for comparison of quantitative values between two groups. The Kruskal–Wallis test was used to compare more than two groups. Tukey’s multiple comparisons test was used after Kruskal-Wallis test to compare each experimental group against each control group. Quantitative data are described as median and range or as mean ± standard deviation. Statistical differences were considered significant if p< 0.05. GraphPad Prism version 9.4.1 for Windows (GraphPad Software, San Diego, CA, USA) and SPSS (version 19.0; SPSS Inc., Chicago, IL, USA) were used to calculate statistical significance.

## 3 Results

### 3.1 AD patients with HND exhibited severe clinical/laboratory phenotypes

Among the 5,007 patients with AD, 120 (2.4%) had clinical records indicating HND. The 120 HND and 4,887 non-HND patients were age- and sex-matched using the exact matching method. After eliminating all missing values in the database, 74 HND and 74 non-HND patients were matched for comparison. There were no significant differences in the distribution of age, sex, and disease onset between the HND and non-HND groups (**Table 1**). Regarding AD phenotypes, there was a higher percentage of extrinsic AD (total serum IgE > 200 IU/mL) in the HND group (**Table 1**). Interestingly, AD patients with HND showed a higher disease severity, as measured by the EASI score (**Table 1**. p<0.0001) and elevated total IgE (**Table 1**. p-value<0.0001) during the initial clinic visit.

**Table 1:** Comparison of epidemiological and laboratory characteristics of HND and Non-HND patients.

Characteristics	HND	Non-HND	*p*-value
No. subjects	74	74	
Age, yrs (range)	24 (10-41)	24 (10-41)	>0.9999
Sex			>0.9999
Male, n (%)	48 (64.9)	48 (64.9)	
Female, n (%)	26 (35.14)	26 (35.14)	
Extrinsic AD, n (%)	71 (95.95)	62 (83.78)	0.0038
Onset of AD, yrs			0.4303
Birth~2 years, n (%)	10 (13.51)	11 (14.86)	
3-6 yrs	11 (14.86)	14 (18.92)	
7-12 yrs	10 (13.51)	11 (14.86)	
13-16 yrs	13 (17.57)	10 (13.51)	
16-20 yrs	8 (10.81)	8 (10.81)	
> 20 yrs	20 (27.03)	18 (24.32)	
EASI, score (mean, range)	24.15 (2.00-58.00)	14.64 (0.30-63.40)	<0.0001
Total IgE, IU/mL (mean, range)	3021.3 (39.00-5000.00)	751.9 (9.19-5000.00)	<0.0001

HND, head and neck dermatitis; Non-HND, non-head and neck dermatitis; AD, atopic dermatitis; EASI, Eczema Area and Severity Index.

Paired Student’s t-test was done for statistical analysis.

### 3.2 Increased sensitization to *M. furfur* in adolescent and adult AD patients with HND

Next, we concentrated on the effect of “fungiome” composition to the HND in AD, especially on *M. furfur*, which has been associated with dandruff (14, 15), HND in AD (4, 16), and AD itself (17). To elucidate the differences in *M. furfur* sensitization status between the HND and non-HND groups, 312 patients were enrolled in the analysis. Of the 312 patients with AD, 75 were in their childhood, 61 were in adolescence, and 176 were adults. The average age of the patients was 24.45 ± 14.56, consisting of 169 male and 143 female AD patients.

**Figure 1:**
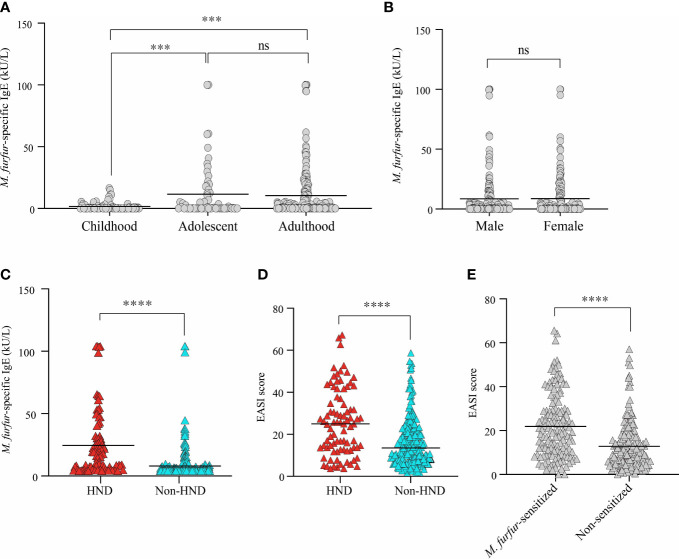
Comparison of serum *M. furfur*-specific IgE level according to **(A)** age groups, **(B)** sex, and **(C)** the presence of HND. Clinical severity according to **(D)** the presence of HND, **(E)** *M. furfur* sensitization status. (Unpaired t-test, **(A)** Childhood vs. Adolescent; p = 0.0002, Adulthood vs. Adolescent; p = 0.7392, Adulthood vs. Childhood; p = 0.0003, **(B)** p = 0.9634, **(C)** p< 0.0001 **(D)** p< 0.0001 **(E)** p< 0.0001) HND, Head and neck dermatitis. *** : p<0.001; **** : p<0.0001; ns, Not Significant (p >0.05).

The average level of IgE specific to *M. furfur* was 8.57 ± 18.89 kU/L. In the childhood group, the specific IgE level was 1.689 ± 3.46 kU/L, and the levels for the adolescence and adulthood groups were 11.53 ± 22.21 kU/L and 10.48 ± 20.8 kU/L, respectively. The difference between adolescents and adults was not significant, whereas the data were significant for both childhood and adolescence and between childhood and adulthood (**Figure 1A**; both p<0.001) The sex-specific level of specific IgE to *M. furfur* was also assessed, and it was found to be 8.53 ± 19.62 kU/L in males and 8.63 ± 18.05 kU/L in females (**Figure 1B**). The difference between the two groups was not statistically significant.

### 3.3 Sensitization to *M. furfur* is associated with increased severity and occurrence of HND

The association between HND and sensitization to *M. furfur* was evaluated. Interestingly, the levels of IgE specific to *M. furfur* was significantly higher in the patients with HND (**Figure 1C**; 20.61 ± 26.04 vs. 4.07 ± 12.80 kU/L, p<0.0001). In addition to laboratory characteristics, the association between the presence of HND and clinical severity, which was measured using the EASI score, revealed that clinical severity was significantly higher in HND patients (**Figure 1D**; 24.12 ± 15.90 vs. 14.28 ± 11.50, p<0.0001).

The patients were categorized according to their sensitization to *M. furfur*. Of the 312 patients, 142 were sensitized and the remaining 170 were non-sensitized. EASI scores of the *M. furfur* sensitized AD patients were significantly higher than those of the non-sensitized group (**Figure 1E**; 21.91 ± 14.59 vs. 12.83 ± 11.07, p<0.0001). These data indicate that sensitization to *M. furfur* not only affects the risk of HND in AD but might also contribute to the overall severity of AD.

### 3.4 Comparison of histopathological characteristics between HND and non-HND subjects

**Figure 2:**
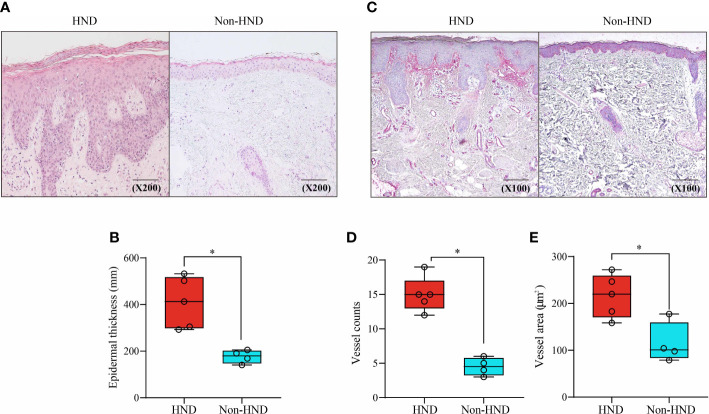
Histopathological comparison of facial skin of HND and non-HND subjects. **(A)** Representative image biopsy specimen of HND (left) and non-HND (right) facial pathology. **(B)** Average epidermal thickness of patients with HND was higher than that of non-HND subjects. (200x magnification) **(C)** Immunohistochemistry of the upper dermis for factor VIIIa-related antigen of patients with (left) and without (right) HND. (200x magnification) **(D)** Average vessel count and average vessel area of patients with HND was significantly higher than that of non-HND subjects. (Mann-Whitney U Test, **(B)** p = 0.0159, **(D)** p = 0.0159 **(E)** p = 0.0317) HND, Head and neck dermatitis. * : p<0.05.

To investigate histological differences between the HND and non-HND groups, skin biopsy specimens were examined by hematoxylin and eosin staining. In HND pathology, a general trend of hyperkeratosis, acanthosis, and parakeratosis was observed, with dense inflammatory cells surrounding the increased vasculature (**Figure 2A**). The average epidermal thickness in HND group (412.5(292.8–532.1) μm) was significantly higher than that of the non-HND group (179.6(140.9–205.3) μm) (**Figure 2B**; p<0.05).

Increased vascularity in HND patients was confirmed by immunohistochemical staining for factor VIII-related antigens (**Figure 2C**). Average vessel count for HND group was 15 (12–19) with a concurrent average vessel area of 221.4 (159.8–273.4) µm^2^. The average vessel count in non-HND histology was 4.5 (3–6) with an average vessel area of 101.0 (78.9–177.3) µm^2^. The differences in vessel counts and average vessel sizes were statistically significant (**Figures 2D, E**; both p<0.05).

**Figure 3:**
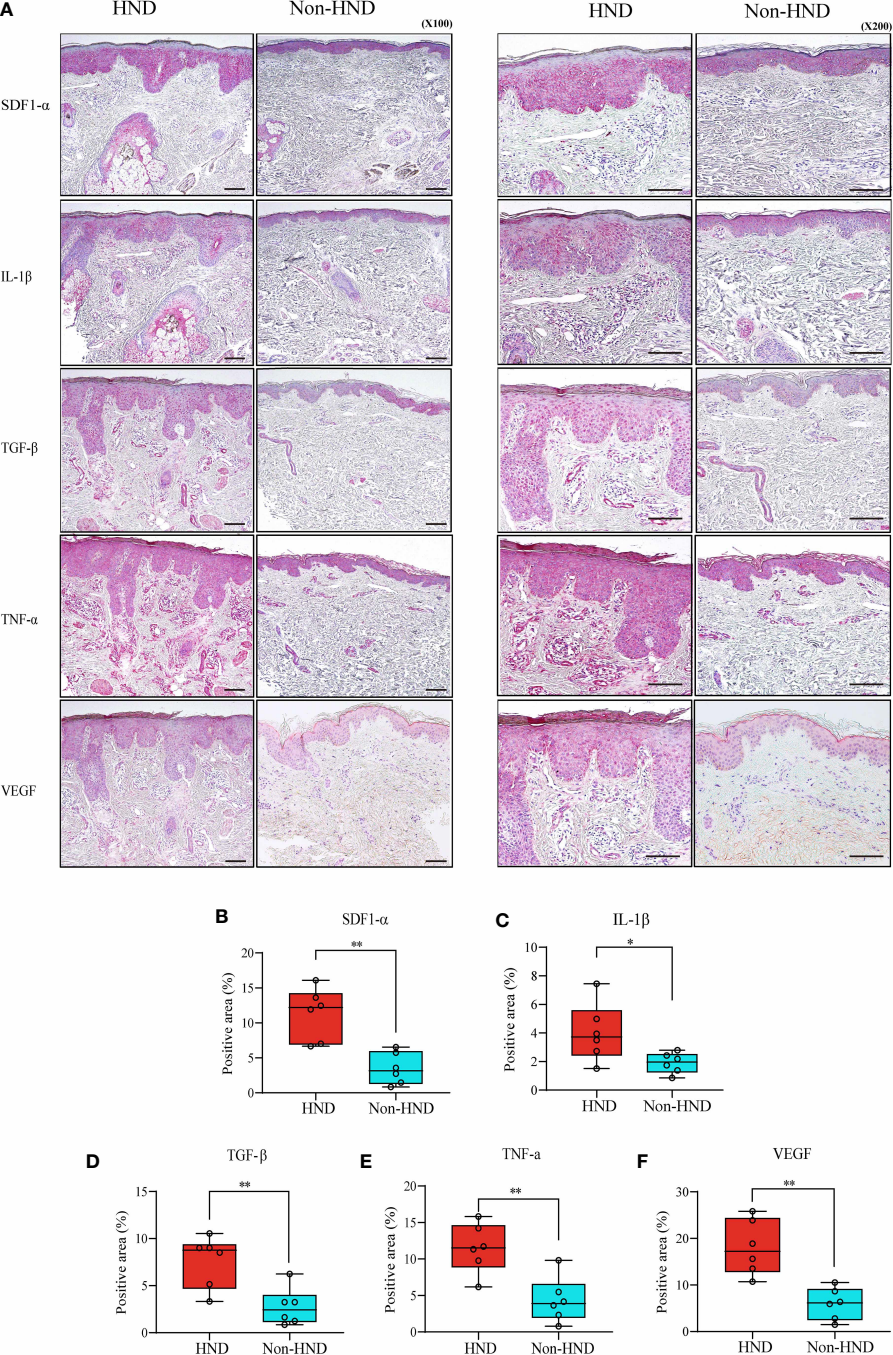
Immunohistochemistry of HND and non-HND facial skin specimens. **(A)** Representative histological image at 100x magnification (left) and 200x magnification (right). Quantified staining intensity of **(B)** SDF-1α, **(C)** IL-1β, **(D)** TGF-β, **(E)** TNF-α, and **(F)** VEGF. (Unpaired two-tailed t-test, SDF-1α; p = 0.0014, IL-1β; p = 0.0374, TGF-β; p = 0.0058, TNF-α; p = 0.0035, VEGF; p = 0.0002) HND, head and neck dermatitis; SDF-1 *α*, stromal cell-derived factor-1-alpha; IL-1 *β*, Interleukin-1-beta; TGF- *β*, transforming growth factor-beta; TNF- *α*, tumor necrosis factor-alpha; VEGF, vascular endothelial growth factor. * : p<0.05; ** : p<0.01.

Next, immunohistochemical staining of pro-inflammatory cytokines and chemokines was performed to confirm the presence of inflammatory mediators in HND lesions. The evaluated molecules included TNF-α, TGF-β, IL-1β, SDF1-α, and VEGF. As a result of IHC staining, all molecules showed significantly higher signal in HND group, confirming that the HND patients’ facial skin show intense inflammatory circumstance than non-HND group. (**Figures 3A–F**; SDF1-α, p<0.01; IL-1β, p<0.05; TGF-β, p<0.01, TNF-α, p<0.01; VEGF, p<0.001),

### 3.5 VEGF, VEGFR, IL-31, and IL-33 levels were upregulated in keratinocytes cultured with *M. furfur*

*Malassezia* spp. colonization/abundance is known to be linked with AD pathogenesis, as AD patients often exhibit hypersensitization to *Malassezia* spp. with higher *Malassezia-*specific IgE levels (18–21). These findings from previous studies indicate the possibility of a positive association between *Malassezia* spp. abundance and hypersensitivity.

**Figure 4:**
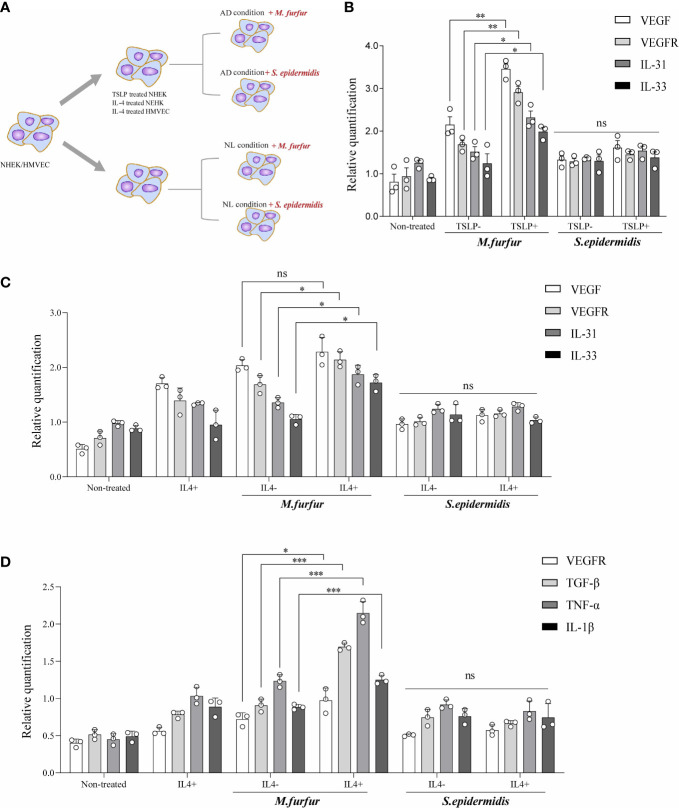
*In vitro* experiments showing **(A)** increased expression levels of VEGF, VEGFR, IL-31, and IL-33 in keratinocytes cultured with *M. furfur*, supplemented with **(B)** TSLP or **(C)** IL-4*. M. furfur* induced increased expression levels of VEGFR, TGF-β, TNF-α, and IL-1β in **(D)** endothelial cells supplemented with IL-4. (three replicates for each column, Unpaired t-test, **(B)** VEGF; p = 0.0044, VEGFR; p = 0.0019, IL-31; p = 0.0145, IL-33; p = 0.0381, **(C)** VEGF; p = 0.1244, VEGFR; p = 0.0244, IL-31; p =0.0382, IL-33; p = 0.0117, **(D)** VEGFR; p-value 0.0266, TGF-β; p-value 0.0002, TNF-α; p-value 0.0009, IL-1β; p-value 0.0009). * : p<0.05; ** : p<0.01; *** : p<0.001; ns, Not Significant (p>0.05).

Thus, the effects of *M. furfur* colonization on skin were evaluated *in vitro* (**Figure 4A**). First, keratinocytes (normal human epidermal keratinocytes) were cultured with either *M. furfur* or *S. epidermidis*, and the expression levels of VEGF, VEGFR, IL-31, and IL-33 were evaluated. To create an environment similar to that of AD, TSLP and IL-4 were used. As shown in **Figure 4B**, the expression levels of VEGF, VEGFR, IL-31, and IL-33 were significantly increased when the cells were co-cultured with *M. furfur* and treated with TSLP (VEGF, p<0.01; VEGFR, p<0.01, IL-31; p<0.05; IL-33, p<0.05). No significant increase in expression was observed in keratinocytes co-cultured with *S. epidermidis*. The expression levels did not increase when cells were not treated with TSLP. As shown in **Figure 4C**, similar results were observed when cells were treated with IL-4, except for VEGF (VEGF, p = 0.1244; VEGFR, p<0.05, IL-31; p<0.05, IL-33; p<0.05). These results suggest that *M. furfur* induces the production of cytokines related to AD, further exacerbating disease severity, and that the expression of VEGF, which induces angiogenesis, may contribute to the erythema observed in HND of AD.

### 3.6 *M. furfur* induced increased expression levels of VEGFR, TGF-β, TNF-α, and IL-1β in endothelial cells

**Figure 4D** depicts the effect of *M. furfur* on endothelial cells. Human microvascular endothelial cells were co-cultured with *M. furfur* and treated with IL-4 using the same protocol as that used for keratinocytes. The increase in the expression levels of VEGFR, TGF-β, TNF-α, and IL-1β was most significant when the endothelial cells were co-cultured with *M. furfur* and treated with IL-4 (**Figure 4D**; VEGFR, p<0.05; TGF-β, p<0.001; TNF-α, p<0.001, IL-1β; p<0.001). However, this tendency was not observed in endothelial cells co-cultured with *S. epidermidis*. These results imply that *M. furfur* also affects endothelial cells, inducing the production of pro-inflammatory cytokines, such as TGF-β, TNF-α, and IL-1β, as well as VEGFR, which further promote vascular proliferation.

### 3.7 Decreased ceramide in AD patients with HND

*Malassezia* spp. are well known for their ability to produce lipases (22) that break down cutaneous lipid components into unsaturated fatty acids, oleic acid, and arachidonic acid (23, 24). Therefore, we investigated the lipid composition of the stratum corneum, which is essential for the maintenance of skin barrier function.

**Figure 5:**
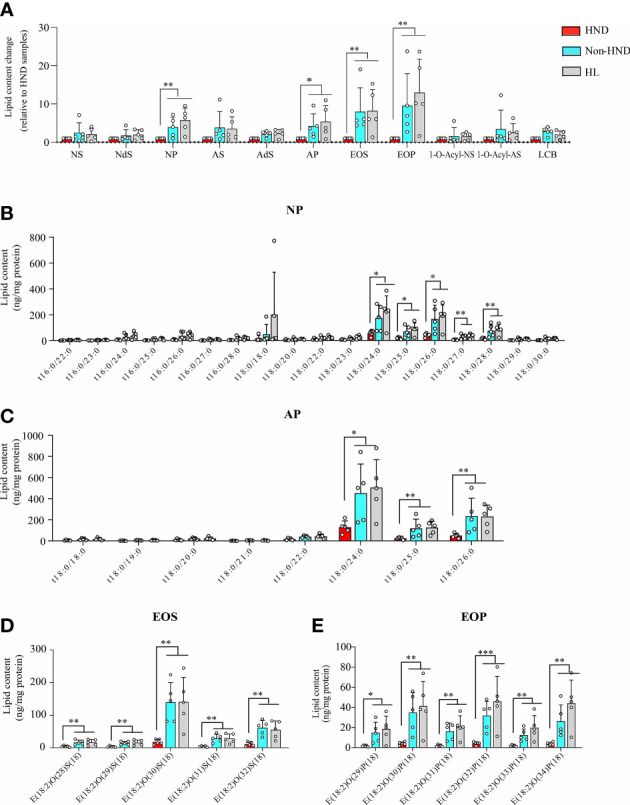
Decreased ceramide in AD patients with HND. **(A)** Overall differences in the amounts of various ceramide types according to the presence of HND. Individual ceramide components belonging to ceramide types, which were significantly reduced in HND patients; **(B)** NP, **(C)** AP **(D)** EOS, **(E)** EOP. (five replicates for each column, Mann-Whitney U Test, **(A)** NP; p = 0.0031, AP; p = 0.0111, EOS; p = 0.0029, EOP; p = 0.0012, **(B)** t18:0/24:0; p = 0.0431, t18:0/25:0; p = 0.0272, t18:0/26:0; p = 0.018, t18:0/27:0; p = 0.0035, t18:0/28:0; p = 0.0029, **(C)** t18:0/24:0; p = 0.0176, t18:0/25:0; p = 0.0021, t18:0/26:0; p = 0.0024, **(D)** E(18:2)O(28)S(18); p = 0.0055, E(18:2)O(29)S(18); p = 0.0055, E(18:2)O(30)S(18); p = 0.0029, E(18:2)O(31)S(18); p = 0.0024, E(18:2)O(32)S(18); p = 0.0024, **(E)** E(18:2)O(29)P(18); p = 0.0105, E(18:2)O(30)P(18); p = 0.0024, E(18:2)O(31)P(18); p = 0.0029, E(18:2)O(32)P(18); p = 0.0009, E(18:2)O(33)P(18); p = 0.0012, E(18:2)O(34)P(18); p = 0.0012) Abbreviations: NP, nonhydroxyacyl phytosphingosine; AP, *α*-hydroxyacyl-phytosphingosine; EOS, esterified ω-hydroxyacyl sphingosine; EOP, esterified ω-hydroxyacyl phytosphingosine; HND, head and neck dermatitis; Non-HND, non-head and neck dermatitis; HL, healthy control. * : p<0.05; ** : p<0.01; *** : p<0.001.

Tape stripping was performed on the facial skin of patients with HND and those without HND, with five subjects in each group. Five additional subjects without any underlying disease were also tested as controls. Patients with HND had significantly higher erythema index and transepidermal water loss levels in the facial area, which supported the presence of HND (**Table 2**). Among the various ceramides from the stratum corneum, phytoceramide NP (nonhydroxyacyl-phytosphingosine), AP (*α*-hydroxyacyl-phytosphingosine), and EO (esterified omegahydroxyacyl) type ceramides (EOS (esterified omega-hydroxyacyl-sphingosine), and EOP (esterified omega-hydroxyacyl-phytosphingosine)) were found to be significantly reduced in the HND group when compared to the non-HND and control groups (**Figure 5A**, NP; p<0.01, AP; p<0.05, EOS; p<0.01, EOP; p<0.01). However, when the non-HND group was compared to the healthy controls, no significant differences were found. Next, individual lipid components belonging to each ceramide type that varied significantly were compared. In the case of NP- and AP-type ceramides, there was individual lipids with no significant difference between non-HND group and healthy controls. Some individual lipid components showed statistical differences between HND group and non-HND/healthy controls (**Figure 5B**, t18:0/24:0; p<0.05, t18:0/25:0; p<0.05, t18:0/26:0; p<0.05, t18:0/27:0; p<0.01, t18:0/28:0; p<0.01, **Figure 5C**, t18:0/24:0; p<0.05, t18:0/25:0; p<0.01, t18:0/26:0; p<0.01). However, all individual lipid components belonging to the EO-type ceramides exhibited statistically significant differences between HND group and non-HND/healthy controls while non-HND group and healthy controls showed no significant difference (**Figure 5D**, E(18:2)O(28)S(18); p<0.01, E(18:2)O(29)S(18); p<0.01, E(18:2)O(30)S(18); p<0.01, E(18:2)O(31)S(18); p<0.01, E(18:2)O(32)S(18); p<0.01, **Figure 5E**, E(18:2)O(29)P(18); p<0.05, E(18:2)O(30)P(18); p<0.01, E(18:2)O(31)P(18); p<0.01, E(18:2)O(32)P(18); p<0.001, E(18:2)O(33)P(18); p<0.01, E(18:2)O(34)P(18); p<0.01).

**Table 2:** Basal characteristics, EI, TEWL, and total serum IgE of study subjects.

Characteristics HND	HND	N-HND	NL	*p-*value
Age, yrs (range)	31 (27-51)	26 (19-28)	31 (22-38)	0.0828
Male, n (%)	2 (40)	4 (80)	4 (80)	0.3263
EI, arbitrary unit (range)	574 (373-617)	320 (274-383)	352 (274-393)	0.0172
TEWL, g/m^2^/h (range)	45 (30-74)	26 (14-31)	19 (9-26)	0.01
Total IgE, IU/mL (range)	2529 (29.9-5000)	2530 (193-5000)	15.7 (8.52-397)	0.0226

EI, erythema index; TEWL, transepidermal water loss; HND, head and neck dermatitis, N-HND, non-head and neck dermatitis, NL, normal.

Kruskal–Wallis test was done for statistical analysis (significant if p<0.05).

**Figure 6:**
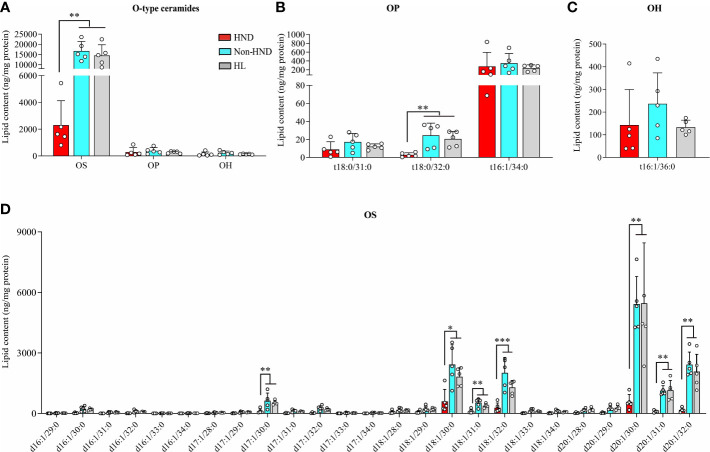
*ω*-Hydroxyceramides (O-type ceramides) contents in human stratum corneum. **(A)** Overall O type ceramides **(B)** OP type ceramides, **(C)** OH (t16:1/36:0) ceramides, and **(D)** OS type ceramides. (five replicates for each column, Mann-Whitney U Test, **(A)** OS; p = 0.0018, OP; p = 0.3833, OH; p = 0.2839, **(B)** t18:0/31:0; p = 0.2516, t18:0/32:0; p = 0.0021, t18:0/34:0; p = 0.538, **(C)** t16:1/36:0; p = 0.2839, **(D)** d17:1/30:0; p = 0.0055, d18:1/30:0; p = 0.0117, d18:1/31:0; p = 0.005, d18:1/32:0; p = 0.0006, d20:1/30:0; p = 0.0029, d20:1/31:0; p = 0.0029, d20:1/32:0; p = 0.0012) Abbreviations: OP, ω-hydroxyacyl phytosphingosine; OH, ω-hydroxyacyl 6-hydroxydsphingosine; OS, ω-hydroxyacyl sphingosine; HND, head and neck dermatitis; Non-HND, non-head and neck dermatitis; HL, healthy control. * : p<0.05; ** : p<0.01; *** : p<0.001.

In the above analysis, the decrease ratio between Non-HND/Control to HND group was more prominent in EO type ceramides (EOS, EOP) than that of other types. Among the four EO type ceramides, EOS ceramide is regarded as the main ceramide component of the epidermis (25). In the epidermis, EOS is converted to *ω*-hydroxyacyl-sphingosine (OS) ceramide. The converted OS is attached to the cornified envelope to maintain the skin barrier function (26–29). Based on the above results and that of previous studies, OS ceramides were analyzed after the extraction of hydrolyzed unbound ceramides. As a result, OS ceramides, which are the most abundant in the stratum corneum, were found to be significantly decreased in the HND group when compared to that in the non-HND and control groups (**Figure 6A**, OS; p<0.01, OP; p = 0.3833, OH; p = 0.2839). In addition, when the individual lipid components were compared among the three groups, statistically significant differences were found mostly in those belonging to OS-type ceramides (**Figure 6B**, t18:0/31:0; p = 0.2516, t18:0/32:0; p<0.01, t18:0/34:0; p = 0.538, **Figure 6C**, t16:1/36:0; p = 0.2839, **Figure 6D**, d17:1/30:0; p<0.01, d18:1/30:0; p<0.05, d18:1/31:0; p<0.01, d18:1/32:0; p<0.001, d20:1/30:0; p<0.01, d20:1/31:0; p<0.01, d20:1/32:0; p<0.01). These data indicate that ceramide levels, especially EOS-type ceramides, are downregulated in HND. Considering the ability of *Malassezia* spp. to produce lipases, it can be inferred that the *Malassezia* spp. colonization might have been involved in the decreased stratum corneum ceramide levels of HND patients.

### 3.8 Inhibitory effect of OS-type ceramide on the colonization of M. furfur

**Figure 7:**
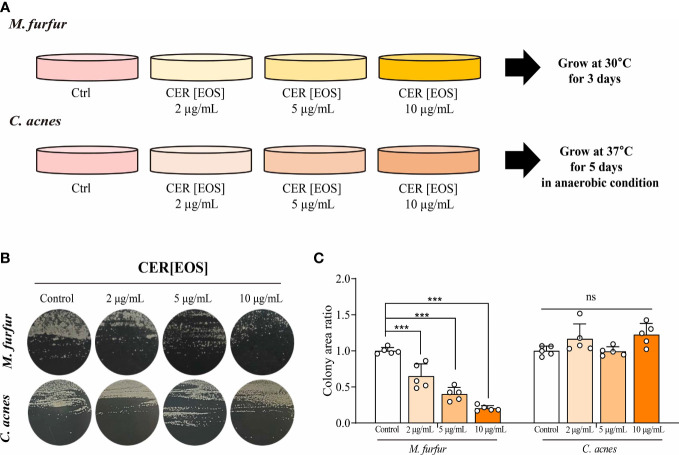
Inhibitory effect of OS-type ceramide on the colonization of *M. furfur.* **(A)** Representative image showing the gross morphology of colonies from (up) *M. furfur* and (down) *C*. *acnes.* **(B)** Quantified area of colonies from (left) *M. furfur* and (right) *C*. *acnes.* (Five replicates for each column, Tukey’s multiple comparisons test; control vs. 2μg/mL; p< 0.001, control vs. 5μg/mL; p< 0.001, control vs. 10 μg/mL; p< 0.001). *** : p<0.001; ns, Not Significant (p>0.05).

Finally, we investigated whether the restoration of OS-type ceramides could reduce the colonization of *Malassezia* spp. *in vitro* (**Figure 7A**). To assess the effect of ceramide on the growth of *M. furfur*, 0, 2, 5, and 10  *μ* g of EOS (esterified ω-hydroxyacyl-sphingosine) ceramide were added to the growth medium of *M. furfur*. *C. acnes*, the most common bacteria in the normal flora of facial skin, was used as a control, and the same experimental protocol was applied. For *C. acnes*, the total area of colonization did not vary with the concentrations of ceramide (**Figure 7B**). In contrast, in the case of *M. furfur*, as the concentration of ceramide increased, the total area of colonization of *M. furfur* decreased significantly (**Figure 7C**, Tukey’s multiple comparisons test; control vs. 2μg/mL; p< 0.001, control vs. 5μg/mL; p< 0.001, control vs. 10 μg/mL; p< 0.001). These data indicate that ceramide, especially EOS ceramide, inhibits the growth of *M. furfur*.

## 4 Discussion

AD is a chronic condition that severely affects patients’ quality of life (30), and among its various clinical features, HND may be one of the most deleterious symptoms, as the facial skin is involved. Even after the emergence of biologics, including dupilumab, head and neck dermatitis is often encountered in patients with AD (31–33). Therefore, it is necessary to explore the influence of skin barrier and microbes on the occurrence of HND, in addition to the direct blocking of the Th2 response.

Previous reports have identified a link between *M. furfur* and HND in AD (34–37), as its specific IgE levels were significantly increased in these patients. To verify this relationship further, *M. furfur*-specific IgE levels of 312 patients were analyzed and similar results were obtained. In addition to previous findings, patients sensitized to *M. furfur* were found to have significantly higher disease severity. In addition, *M. furfur*-specific IgE levels significantly increased in adolescents and adults compared to pediatric patients, correlating with the increase in subjects with HND after adolescence.

Histopathological studies of adult AD patients with recalcitrant facial erythema revealed a mixture of eczematous and steroid-induced rosacea-like changes (38). Similarly, histological observations in our study revealed hyperkeratosis, acanthosis, parakeratosis, and increased vessels in HND lesions. An increase in the number and average area of vessels in the dermis, confirmed with factor VIII-related antigen, most probably underlies the intense redness noted in patients with HND. Along with the increased vessel counts and vessel area, a higher erythema index indicates that the increase in dermal vasculature may be closely associated with the development of HND in AD patients.

Although a possible link between *M. furfur* and HND has been suggested by various clinical/epidemiological/laboratory studies, the specific mechanism underlying its pathogenesis is largely unknown, with few *in vitro* experimental studies. To determine the effect of *M. furfur*, keratinocytes were co-cultured with *M. furfur* and the expression levels of various cytokines were assessed. Furthermore, as AD is Th2-mediated (39), cells were treated with the associated cytokines, TSLP and IL-4, and compared. Keratinocytes co-cultured with *M. furfur* and treated with either TSLP or IL-4 exhibited significantly increased expression levels of VEGF, VEGFR, IL-31, and IL-33. IL-31 is a cytokine involved in AD that is predominantly associated with pruritus (40), and *M. furfur* has been found to enhance its production, which may contribute to augmented pruritic symptoms. IL-33 is mainly produced by keratinocytes and is associated with the pathogenesis of AD, including tissue remodeling and fibrosis in chronic AD (41). In addition, a recent study indicated that the sebum-microbial metabolite-IL-33 axis may play a role in initiating atopic dermatitis (42). *M. furfur* induces the expression of these cytokines in keratinocytes, which is further enhanced by the Th2 milieu in AD, which may contribute to the exacerbation of the disease (43).

To assess whether endothelial cells were also affected by *M. furfur*, a similar experiment was performed on endothelial cells. *M. furfur*, under the influence of Th2 cytokines, enhanced the production of VEGFR, IL-1β, TNF-α, and TGF-β in HMVEC. Increased expression level of VEGFR leads to increased vascular proliferation and expression of pro-inflammatory cytokines, including IL-1β, TNF-α, and TGF-β, leading to increased inflammatory responses. *M. furfur*, under the influence of Th2 cytokines, leads to angiogenesis and an increase in AD-related cytokines as well as other inflammatory cytokines that may contribute to the exacerbation of skin symptoms.

Skin barrier defects are a crucial part of the pathogenesis of AD and are characterized by decreased ceramide levels in the stratum corneum (44). To determine any possible differences in the skin barrier between AD patients with and without HND, tape stripping was performed on facial skin. Our study indicated that the levels of ceramides were reduced, especially those of EOS, and EOP, in patients with HND compared to those without HND. Ceramides, especially EOS ceramides, which link corneocytes and extracellular lipids, form the extracellular lipid envelope in the stratum corneum and are crucial for the repair of the skin barrier (45, 46). This confirms an additional skin barrier defect in AD patients with HND, which may be associated with the development of HND in AD. Also, the decrement of ceramides might lead to the increased fatty acids in epidermis. These free fatty acids from ceramides might be able to induce chronic inflammation with the aids of fatty acid binding proteins (FABPs) which is known to be associated with Th17 inflammation (47), especially in atopic dermatitis and psoriasis (48, 49). Th17 inflammation is well known for their anti-fungal activities (50–52), so the relationship between the decreased ceramides level in the facial skin of HND patients, FABPs, and Th17 immune response might have to be explored in the near future.

As the decrease in ceramide levels was confirmed in HND, its possible association with the colonization of *M. furfur* was assessed. The growth of *M. furfur* on the culture medium was evaluated based on the number of colonies formed, and different concentrations of EOS ceramide were added to evaluate whether the growth of *M. furfur* was affected by the addition of ceramide. *C. acnes*, a representative species that constitutes the normal flora of the skin, especially the face, was used as a control. As seen in the results, the number of colonies of *M. furfur* significantly decreased with increasing concentrations of EOS ceramide. However, the growth of *C. acnes* was not altered by ceramide. Ceramide levels are lowered in the facial skin of AD patients with HND, and this decrease may enhance the growth and colonization of *M. furfur*.

Some limitations of our study include the limited number of subjects enrolled in lipid analysis from stratum corneum by LC-MS/MS. The statistical methods did not consider the normality of the data due to the low number of samples; hence, further studies might be needed to confirm these data. Moreover, the effect of ceramide on the colonization of *M. furfur* was verified *in vitro*. Further research might be needed to confirm these findings with an *in vivo* AD mouse model and clinical trials with human subjects. Finally, the effect of the microbiome on the development of HND was not evaluated. Especially, *Staphylococcus* species (including *Staphylococcus aureus* and *Staphylococcus epidermidis*) are well-known skin commensal bacteria engaged in the development/exacerbation of AD. Therefore, the relationship between HND and mycobiome (especially *M. furfur*) from our study needs to be interpreted carefully.

**Figure 8:**
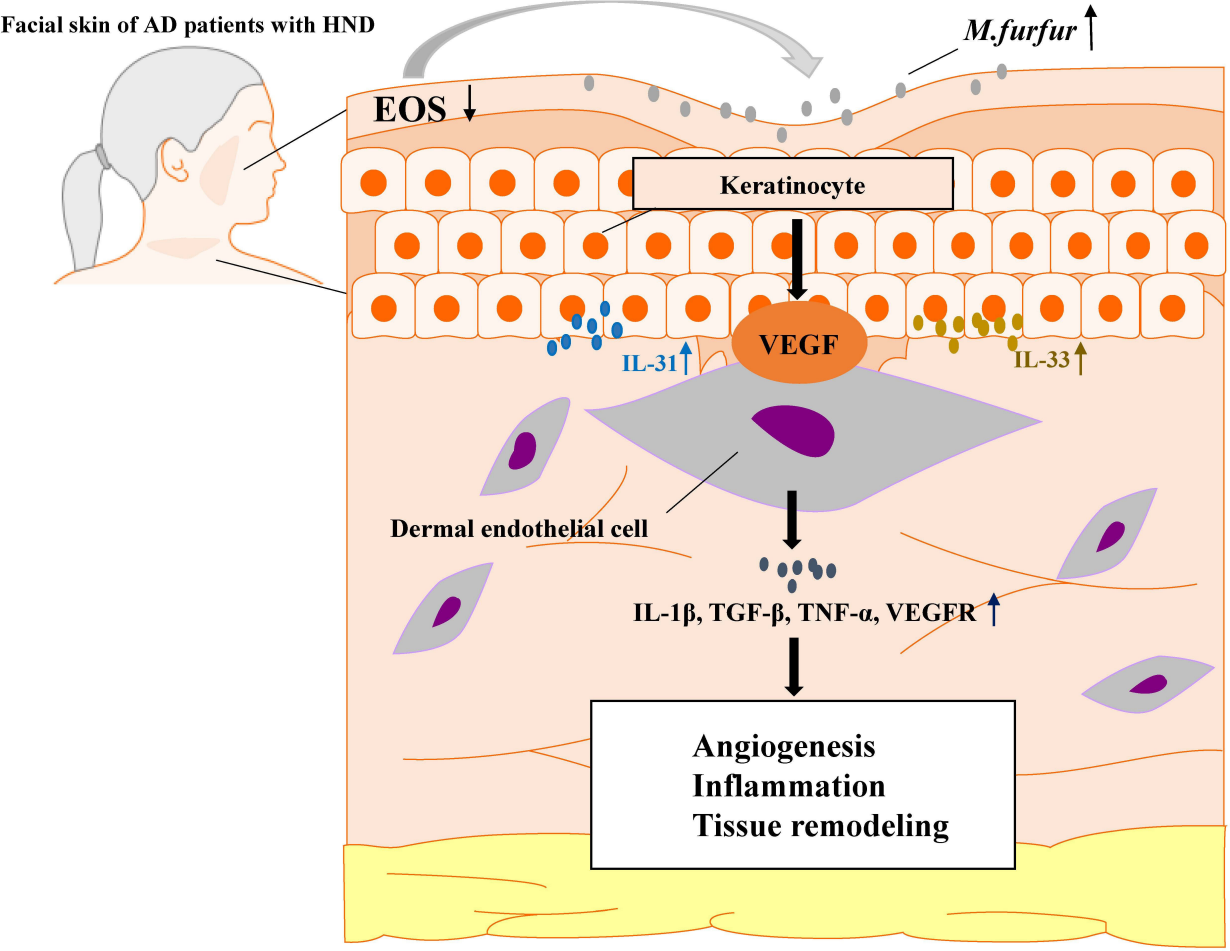
A schematic graph of one pathogenetic flow of HND development in AD patients. *M. furfur* proliferates in the facial skin of patients suffering from HND due to decreased levels of ceramide, which induce keratinocytes to secrete Th2 cytokines (IL-31, IL-33) and vascular endothelial growth factor (VEGF), thereby causing dermal endothelial cells to produce various cytokines to induce angiogenesis, exacerbating eczematous inflammation and tissue remodeling.

Thus, *M. furfur* proliferates in the facial skin of patients suffering from HND due to decreased levels of ceramide, which inhibits *M. furfur* growth. As *M. furfur* proliferates, it induces keratinocytes to produce various cytokines, which are further enhanced by the Th2 milieu of AD as well as other factors, including VEGF, leading to vascular proliferation. This leads to enhanced production of various pro-inflammatory cytokines and vascular proliferation, resulting in exacerbation of the disease (**Figure 8**). Therefore, levels of IgE specific to *M. furfur* may be evaluated in AD patients with refractory HND.

## Data availability statement

The original contributions presented in the study are included in the article/supplementary material. Further inquiries can be directed to the corresponding authors.

## Ethics statement

The studies involving human participants were reviewed and approved by Institutional Review Board of Yonsei University Severance Hospital. Written informed consent to participate in this study was provided by the participants’ legal guardian/next of kin.

## Author contributions

KL, CP, and HC conceived the project and design the study. HC, SMK, KZ, ZW, HL, JK, SHK, HK, WK, and YK collected the data and performed the experiments. HC and SMK wrote the first version of the manuscript. HC, KZ, SMK, and ZW integrated the data and made figures. K-HL supervised a lipid analysis in the skin. YL supervised a mycobiome analysis. CP and K-HL directed the whole experiments and edited the manuscript. All authors contributed to the article and approved the submitted version.
